# The Importance of Clinically Correlating Tuberculosis With Indeterminate QuantiFERON Gold Test

**DOI:** 10.7759/cureus.60282

**Published:** 2024-05-14

**Authors:** Krishna Sheth, Ajith Saju, Meenu Mundackal, Neil Srinivas, Aditya Agarwal

**Affiliations:** 1 Internal Medicine, Garnet Health Medical Center, Middletown, USA; 2 Internal Medicine, Touro College of Osteopathic Medicine, Middletown, USA

**Keywords:** false negative quantiferon, quantiferon tb, tuberculosis, disseminated miliary tuberculosis, quantiferon gold plus, reticulonodular infiltrate, quantiferon tb gold

## Abstract

Tuberculosis (TB) is a highly contagious airborne infection of the lungs. It can present in active form, as well as latent form. The clinical manifestations of tuberculosis can present as either subacute or chronic. Some symptoms include weight loss, night sweats, fevers, and hemoptysis. This case highlights the importance of clinical judgment and follow-up testing when patient presentation does not correlate with initial results. We share a perplexing encounter where a 34-year-old male presented with hemoptysis, fevers of unclear origin, and an indeterminate QuantiFERON Gold result and was empirically started on rifampin, isoniazid, pyrazinamide, and ethambutol (RIPE) therapy. RIPE therapy includes the gold standard medications used to treat tuberculosis.

## Introduction

Tuberculosis (TB) is caused by the acid-fast bacillus *Mycobacterium tuberculosis*. A severe form of tuberculosis is miliary tuberculosis. In this condition, bacteria spread via the blood to affect multiple organ systems [1]. Screening for TB can be done via skin testing or blood testing with the QuantiFERON Gold. The QuantiFERON Gold test exhibits a remarkable specificity of 98.44% in low-risk individuals, indicating its high accuracy in correctly identifying those without latent tuberculosis infection [2]. Moreover, it demonstrates a combined sensitivity of 94.09% in individuals with active disease in the USA, Japan, and Australia with 88.7% being specific to the USA [2]. This signifies its effectiveness in detecting tuberculosis in individuals with an active infection but to a lesser degree. These screening tests are also usually accompanied by chest X-ray, acid-fast stain, and culture. Given the high sensitivity and specificity of the test, it is used as a gold standard for diagnosing and guiding treatment for tuberculosis.

## Case presentation

A 34-year-old male with no pertinent past medical history presented with a chief complaint of hemoptysis. Of note, he immigrated from Ecuador to New York two years prior to admission and denied any recent travel history. He denied a smoking history and exposure to toxins/fumes. The onset of hemoptysis was six months prior to arrival and was accompanied by undulating fever, chills, night sweats, generalized malaise, unintentional weight loss, and shortness of breath. Around the time of onset, he had made two visits to another ED and was diagnosed with COVID-19 pneumonia and discharged home.

**Figure 1:**
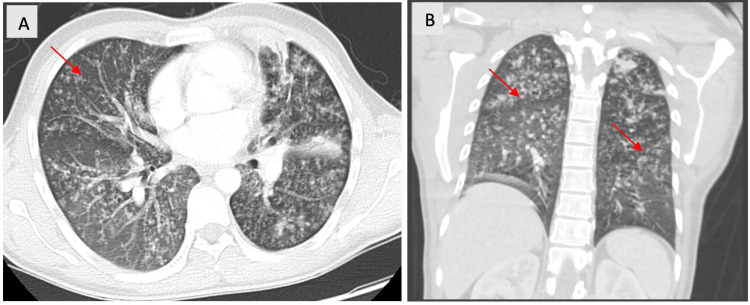
Axial (A) and coronal (B) view of the thorax showing bilateral reticulonodular infiltrates with a confluent infiltrates (red arrows).

On presentation to the emergency room, the patient was febrile and tachycardic and had a leukocytosis of 11.4. Further laboratory results indicated an elevated C-reactive protein of 14.5 and an erythrocyte sedimentation rate of 86, indicating an inflammatory process. Chest X-ray and CT of the thorax supported bilateral reticulonodular infiltrates with a confluent infiltrate more prominent in the left lung (Figure 1). On examination, he appeared fatigued and diaphoretic. The lungs were clear bilaterally without crackles. No enlarged lymph nodes were palpated, and rashes were not observed. He was admitted to the hospital initially for sepsis due to an unclear etiology; however, he failed a course of vancomycin and piperacillin/tazobactam. Despite receiving broad-spectrum antibiotics, he had persistent fevers.

Initially, a QuantiFERON Gold and a mycobacterium culture were ordered due to a suspicion of miliary TB from an endemic country. The QuantiFERON Gold was indeterminate, so a second QuantiFERON Gold was ordered, which yielded a positive result. Several days later, a sputum culture resulted positive for acid-fast bacillus. Fungus culture came back positive for *Candida albicans*. HIV, *Pneumocystis jiroveci* pneumonia (PJP), *Legionella*, *Toxoplasma*, antinuclear antibody, rheumatoid factor, antineutrophil cytoplasmic antibody (ANCA), *Histoplasma*, and *Cryptococcus* all resulted in negative. *Cytomegalovirus* IgG resulted positive, while IgM was negative, signifying a prior resolved infection and no current infection.

The patient was ultimately diagnosed with miliary tuberculosis. He was educated on these findings, and treatment protocol included rifampin, isoniazid, pyrazinamide, and ethambutol (RIPE), with two weeks received inpatient. He was discharged on by mouth (PO) RIPE (rifampin, 600 mg; isoniazid, 330 mg; pyrazinamide, 1000 mg; and ethambutol, 800 mg) for an additional six weeks. Following this, he was informed that he would need to continue rifampin and isoniazid for six months. The patient was advised to take vitamin B6 while taking isoniazid and was encouraged to check monthly liver function tests (LFTs), have ophthalmology checks, and follow up outpatient with infectious disease.

## Discussion

There are several methods of screening for TB including the tuberculin skin test, QuantiFERON, Wantai TB interferon-gamma release assay (IGRA), and T-Spot. The tuberculin skin test elicits a type IV hypersensitivity response after injecting tuberculin material intradermally into the forearm [3]. After 48-72 hours, patients will return to have their skin reaction be read by a qualified healthcare professional. Cutoffs for the induration vary depending on risk factors. Patients who are immunosuppressed, HIV-positive, transplant recipients, or exposed to contact with TB are positive with a skin reaction of >5 mm^2^ [4]. The cutoff for patients from high-risk developing countries and nursing homes, children, and healthcare workers is >10 mm. For low-risk individuals, a reaction of >15 mm is considered positive. The tuberculin skin test can have false positives due to prior Bacillus Calmette-Guérin (BCG) vaccination, exposure to nontuberculous mycobacteria, and the variability in interpreting skin indurations. Sometimes, a two-step skin test will be performed to optimize the accuracy and reliability of TB screening, particularly in populations with a higher risk of TB exposure or in settings where TB transmission is a concern. It helps differentiate between boosted reactions from past exposure and true recent infections.

The following three screening methods are IGRA, QuantiFERON Gold Plus, and Wantai TB-IGRA. IGRAs are blood tests that record T cell response [4]. The QuantiFERON Gold Plus is composed of two TB antigens, including early secreted antigenic target 6 kDa (ESAT6) and 10 kDa culture filtrate protein (CFP10) [4]. Four tubes are drawn from individuals: the first two tubes contain one antigen each, a positive control, and a negative control. Wantai TB-IGRA is comparable to QuantiFERON but only consists of three tubes: one tube containing a recombinant peptide of both antigens, a positive control, and a negative control [4]. T-Spot differs in that peripheral blood mononuclear cells are isolated and incubated with TB antigens prior to measuring interferon (IFN)-γ release. The specificity of the QuantiFERON Gold in comparison to the other IGRAs has not been well established for the long term [5]. IGRAs offer several advantages over the tuberculin skin test, including greater specificity, no need for a follow-up visit to interpret results, and reduced likelihood of false positives due to prior BCG vaccination [6]. However, like any diagnostic test, IGRAs are not ideal and can yield indeterminate results in some cases, as seen in the presented case report. Another disadvantage that should be considered is the poor sensitivity of this test in certain populations, including those with HIV and children.

In the case report, the patient initially had an indeterminate QuantiFERON Gold result, which was subsequently followed up with a second test yielding a positive result. This highlights the importance of clinical judgment and follow-up testing when initial results are inconclusive. This screening tool is a widely used test known for its high specificity and therefore brings into question whether the current standard of care is proficient enough.

Treatment for tuberculosis typically involves a combination of antibiotics, RIPE, taken for several months to effectively eradicate the bacteria and prevent recurrence. Patient education and close follow-up are essential components of tuberculosis management to ensure adherence to treatment, monitor for adverse effects, and prevent the development of drug resistance.

## Conclusions

Tuberculosis remains a significant global health concern, and early detection, accurate diagnosis, and appropriate treatment are crucial for the successful management and control of the disease. Screening tests such as QuantiFERON Gold play a valuable role in identifying individuals with latent or active tuberculosis infection, but clinical judgment and follow-up testing are essential for accurate diagnosis and management, particularly in cases with indeterminate results or atypical presentations.
